# Targeting Cancer Cells with Reactive Oxygen and Nitrogen Species Generated by Atmospheric-Pressure Air Plasma

**DOI:** 10.1371/journal.pone.0086173

**Published:** 2014-01-21

**Authors:** Hak Jun Ahn, Kang Il Kim, Nguyen Ngoc Hoan, Churl Ho Kim, Eunpyo Moon, Kyeong Sook Choi, Sang Sik Yang, Jong-Soo Lee

**Affiliations:** 1 Department of Life Science, Ajou University, Suwon, Korea; 2 Department of Electrical and Computer Engineering, Ajou University, Suwon, Korea; 3 School of Medicine, Ajou University, Suwon, Korea; University of Kentucky, United States of America

## Abstract

The plasma jet has been proposed as a novel therapeutic method for cancer. Anticancer activity of plasma has been reported to involve mitochondrial dysfunction. However, what constituents generated by plasma is linked to this anticancer process and its mechanism of action remain unclear. Here, we report that the therapeutic effects of air plasma result from generation of reactive oxygen/nitrogen species (ROS/RNS) including H_2_O_2_, Ox, OH^−^, •O_2,_ NOx, leading to depolarization of mitochondrial membrane potential and mitochondrial ROS accumulation. Simultaneously, ROS/RNS activate c-Jun NH_2_-terminal kinase (JNK) and p38 kinase. As a consequence, treatment with air plasma jets induces apoptotic death in human cervical cancer HeLa cells. Pretreatment of the cells with antioxidants, JNK and p38 inhibitors, or JNK and p38 siRNA abrogates the depolarization of mitochondrial membrane potential and impairs the air plasma-induced apoptotic cell death, suggesting that the ROS/RNS generated by plasma trigger signaling pathways involving JNK and p38 and promote mitochondrial perturbation, leading to apoptosis. Therefore, administration of air plasma may be a feasible strategy to eliminate cancer cells.

## Introduction

Plasma is often referred to as the fourth state of matter in addition to solid, liquid, and gas. Plasma is quite similar to gas in which a proportion of the particles is ionized and charged, some particles are electrically neutral, and some are chemically activated radicals. Plasma can be categorized as either “thermal (or hot) plasma” or “non-thermal (or cold) plasma.” Numerous techniques using plasma have been investigated and successfully implemented in certain industrial applications. Recently, plasma applications have been employed in biological and medical sciences, including blood coagulation [1], cancer therapy [2], surface sterilization [3], and dental cavity treatment [4]. In particular, increasing plasma translational research in cancer treatment may promise novel therapeutic effects. Moreover, it is interesting that cold plasma generated at atmospheric pressure increases the feasibility of medical applications.

Although the biologically effective material(s) generated from plasma and its cellular targets remain unknown, several lines of evidence link reactive oxygen/nitrogen species (ROS/RNS) to its biological effects. Treatment with plasma caused the depolarization of mitochondrial membrane potential and generation of ROS in human cells [2]. In addition, antioxidants ameliorated plasma-induced mitochondrial dysfunction, supporting the notion that oxidizing species such as ROS may mediate plasma-induced effects on mammalian cells [2]. A wide range of seemingly unrelated and complex causes of mitochondrial dysfunction have common underlying pathophysiological mechanisms: ROS production and accumulation of mitochondrial damage, leading to increased oxidative stress, loss of ATP, mitophagy for quality control and elimination of damaged mitochondria, and eventually cell death [5].

It is now clear that ROS have a cell signaling role in many biological systems from bacteria to mammalian cells [6]. ROS can activate cell signaling cascades, such as those involving many different mitogen-activated protein kinase (MAPK) cascades [7], [8]. These include the stress kinases, c-Jun N-terminal kinases (JNK) and stress-activated protein kinase (SAPK). JNKs were identified as a kinase that binds and phosphorylates c-Jun on Ser-63 and Ser-73 within its transcriptional activation domain. JNK is activated by the treatment of cells with cytokines (e.g., tumor necrosis factor (TNF) and interleukin (IL)-1) and by the exposure of cells to many forms of environmental stress (e.g., osmotic stress, redox stress, and radiation) [9]. It has been well established that ROS are potent inducers of JNK. Most reports on ROS-induced JNK activation result from exogenous ROS, mostly H_2_O_2_. In addition, ROS accumulation induced by apoptotic stimuli can activate the SAPK p38 [10]. The JNK and p38 MAPK pathways share several upstream regulators, and accordingly there are multiple stimuli that simultaneously activate both pathways.

Links between ROS signaling and apoptosis are proposed to be mediated by mitochondria. Currently many studies have suggested that mitochondria are the main site of action for JNK in apoptosis. Both JNK1- and JNK-deleted primary murine embryonic fibroblasts have shown resistance to UV-induced apoptosis due to a defect in the mitochondrial death signaling pathway, including failure to release cytochrome c [11]. Mitochondrial translocation of JNK occurs in conditions of stress such as exposure to UV, ionizing radiation, ROS and RNS, and thus mitochondrial-localized JNK provides proximity to mitochondria-generated ROS [12], [13]. In addition, apoptotic stimuli sometimes trigger p38α activation by a secondary route, such as the production of ROS [13].

We and several other groups have reported that atmospheric-pressure plasma [2], [14]–[19] and nitrogen plasma jets from a micro-nozzle array [20] induce apoptosis in cancer cells. Recently, plasma has been shown to promote the apoptosis of cancer cells by the generation of ROS, disruption of the mitochondrial membrane potential, and activation of caspase family proteins [2]. However, what component(s) generated by air plasma produces its anticancer effects and the molecular mechanism by which plasma induces apoptosis remain unclear. For this report, we attempted to find the effector component(s) generated from air plasma and its target and to elucidate the molecular mechanism and biochemical pathway by which it induces anticancer therapeutic benefits. We identified the ROS/RNS as key effectors of air plasma, which target mitochondria and leads to the activation of JNK and p38 signaling pathways.

## Results

### Air micro plasma jet can generate reactive oxygen and nitrogen species

**Figure 1:**
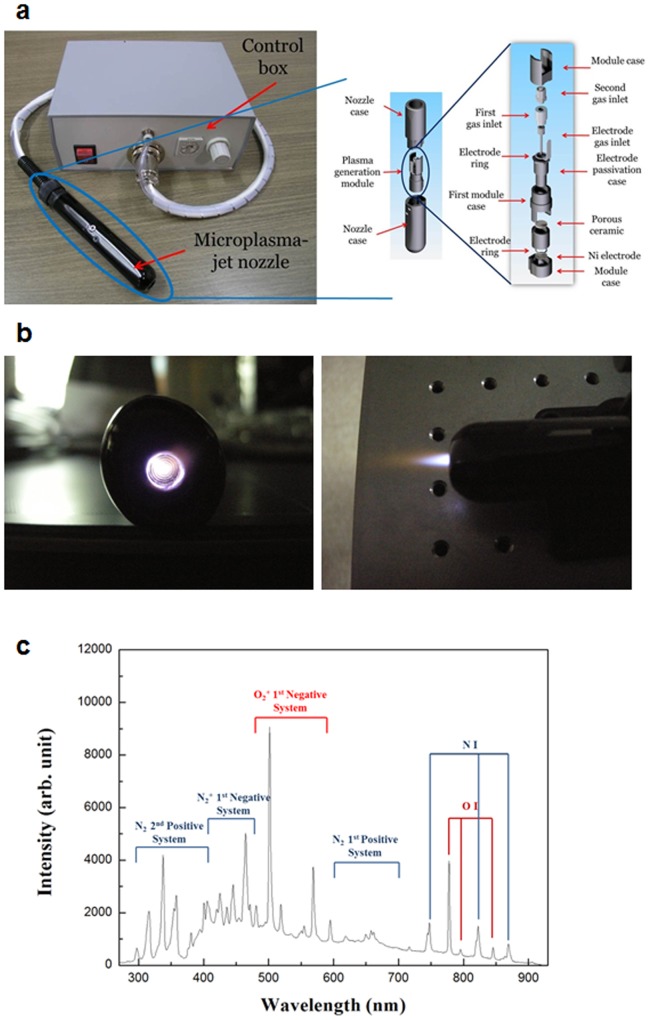
The microplasma jet system and its optical emission spectrum. (a) Photograph of the fabricated microplasma jet system. The inset is a schematic presentation of the micro plasma jet nozzle. (b) Photograph of air micro plasma jet generated at atmospheric pressure. (c) Optical emission spectrum of air micro plasma jet during discharge.

The micro plasma jet system operated at atmospheric pressure can produce chemically active species, particularly oxygen and nitrogen atoms [21]. In this study, we first employed optical emission spectrum analysis to identify the particles and radicals generated by the air plasma system (Figure 1a–b), which can mediate the effects of air plasma in cancer cells [2], [22]. Optical emission spectroscopy (OES) was performed over a wide range of wavelengths from 280 nm to 920 nm with an optical emission spectroscope (SV 2100, K-MAC) (Figure 1c). The dominant emission lines illustrate the presence of excited oxygen ions (O_2_^+^) at 500–600 nm and atomic oxygen (O I) at 777 and 844 nm (Figure 1c). Additionally, the detected reactive species associated with nitrogen are excited nitrogen molecules (N_2_ second positive system and N_2_ first positive system) in the ranges of 300–390 and 610–710 nm and ionized nitrogen molecules (N_2_^+^ first negative system) in the range of 390–480 nm, and atomic nitrogen (N I) at 747, 822, and 868 nm (Figure 1c). These OES results indicate that the air micro plasma jet can play an important role in producing various reactive oxygen and nitrogen species (ROS and RNS, respectively).

### Extracellular and intracellular ROS and RNS were generated by air plasma exposure

**Figure 2:**
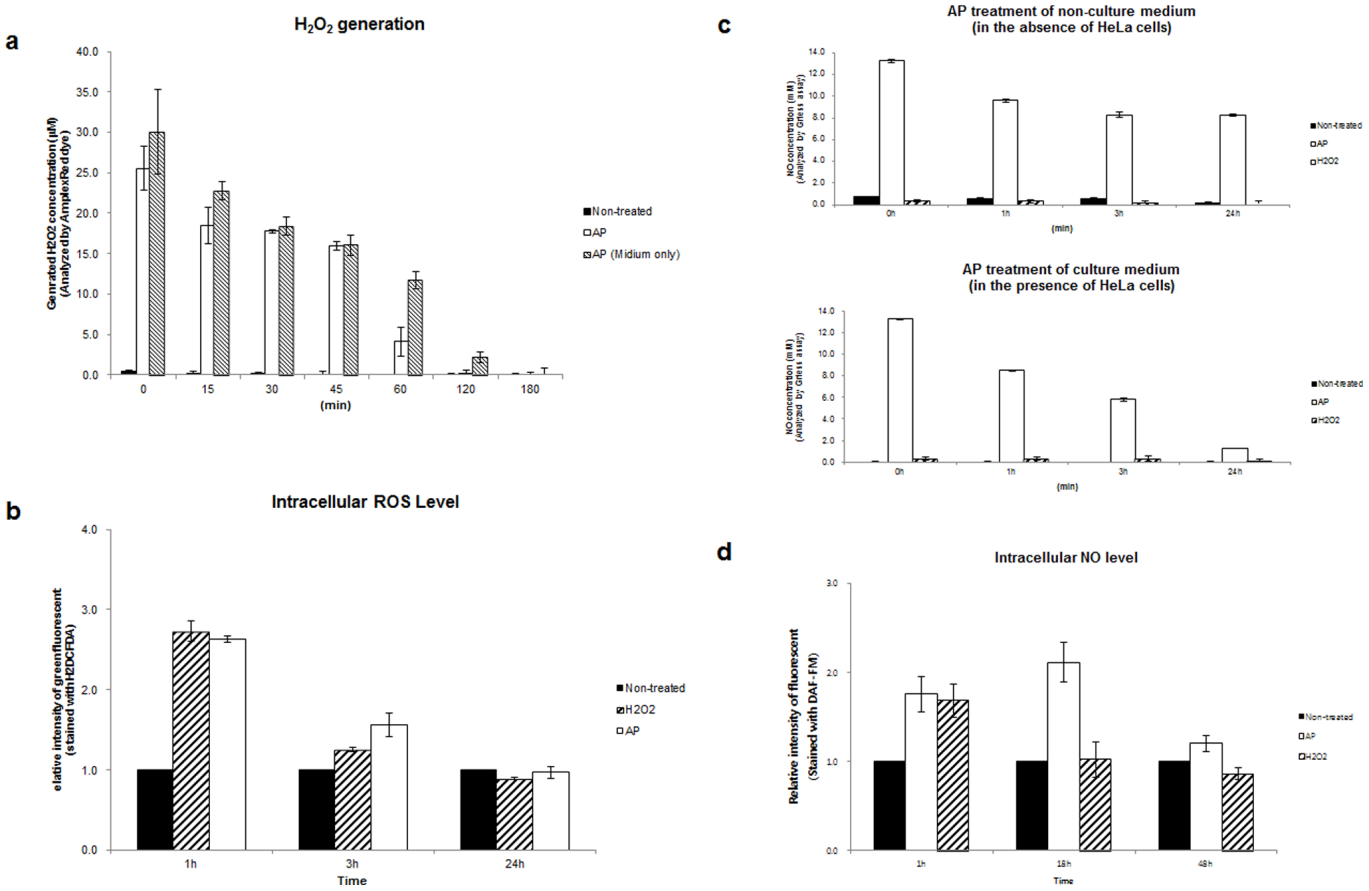
Generation of ROS and RNS by air plasma. (a) Levels of extracellular H_2_O_2_ were determined in culture or non-culture (Medium only) supernatants, with (AP) or without (Non-treated) air plasma treatment. The culture supernatant was harvested at the indicated times following air plasma treatment (AP). AP 0 (min) indicates supernatant harvested immediately after plasma treatment. The concentration of H_2_O_2_ in the culture supernatant was determined by comparison to a H_2_O_2_ standard calibration curve, following incubation with Amplex UltraRed reagent (*n* = 5). (b) Generation and/or penetration across the plasma membrane of ROS including H_2_O_2_, OH^−^, and •O_2_ in the intracellular matrix following air plasma jet (AP) were determined using the ROS-sensitive probe H_2_DCFDA (*n* = 5). The fluorescence of untreated cells (Non-treated) was arbitrarily set to 1. (c) Levels of extracellular NO were determined in non-culture (in the absence of HeLa cells, upper) and culture (in the presence of HeLa cells, bottom) supernatants at the indicated times after air plasma jet exposure by the Griess assay (*n* = 10). (d) Levels of intracellular NO were evaluated using DAF-FM. Data are shown as the mean ± S.E.M. (*n* = 10).

A hint that ROS and RNS may mediate the anticancer effects of air plasma was suggested by our previous study reporting that the air plasma induced apoptosis, accompanied by the generation of ROS and RNS and decreasing mitochondrial membrane potential (MMP) in cervical cancer cells [2]. Moreover, the production of ROS and RNS by plasma jet has been studied recently [23], and is consistent with the emission spectra induced by air plasma (Figure 1c). Therefore, we biochemically analyzed the production of ROS and RNS, their delivery into the liquid phase of the cell culture medium, and the sequential migration of ROS and RNS into cells (Figure 2).

We first examined whether treatment with air plasma would induce the generation of H_2_O_2_ in the culture supernatant (Figure 2a). The culture supernatant was harvested at the indicated times following air plasma treatment and the concentration of H_2_O_2_ in the culture supernatant was analyzed by comparison to H_2_O_2_ standard calibration curve. H_2_O_2_ was generated at approximately 26.93 μM immediately after treatment with air plasma jets and the concentration in the culture supernatant rapidly decreased within 1 h. However, H_2_O_2_ in non-culture medium alone decreased more slowly until 1h following plasma treatment, compared with the level in culture supernatant. In 3 h, the levels of H_2_O_2_ were similar in supernatants from HeLa cell culture and in medium only. H_2_O_2_ was barely detected in the culture supernatant, in 3 h following plasma treatment (Figure 2a). These results suggest that air plasma can generate and sequentially deliver H_2_O_2_ into the medium. In addition, the reason for the initial rapid decrease of H_2_O_2_ generated by air plasma in the culture supernatant compared with that in non-culture medium might be that cells promote scavenging of H_2_O_2_. To test this possibility, we monitored the levels of H_2_O_2_ in culture supernatant and non-culture medium alone following H_2_O_2_ treatment (Figure S1a). When HeLa cells were treated with 2 mM H_2_O_2_, H_2_O_2_ was similarly detected in both culture supernatant and non-culture medium immediately after treatment. However, in 3 h following H_2_O_2_ treatment, H_2_O_2_ was barely detected (approximately 100 μM) in the culture supernatant, but the level remained at approximately 670 μM in non-culture medium (Figure S1a). Based on this result, it is speculated that cells can scavenge H_2_O_2_ generated by air plasma, leading to an accelerated decrease in H_2_O_2_ in the culture supernatant.

We next monitored the generation of intracellular ROS including H_2_O_2_, hydroxyl radicals (OH^−^), and singlet oxygen (•O_2_), following plasma treatment, using H_2_DCFDA (Figure 2b). Following air plasma treatment, intracellular ROS levels increased by approximately 2.7-fold (2.73±0.13). This elevated level decreased until 3 h after plasma treatment and recovered almost to the non-treated basal level in 24 h (Figure 2b). These data suggest that plasma treatment induces the generation of intracellular ROS.

Next, we evaluated whether air plasma may result in the generation of extracellular and intracellular RNS (Figure 2c and d, respectively). Immediately after plasma treatment, extracellular NO levels were elevated to approximately 13.28 mM in both non-culture and culture media (Figure 2c). By 3 h following plasma treatment, extracellular NO levels in non-culture and culture media rapidly decreased to approximately 8.26 or 5.8 mM, respectively (Figure 2c). At time points later than 3 h, the extracellular NO level in non-culture medium was maintained, while the extracellular NO level in culture medium was continuously reduced to approximately 1.28 mM in 24 h following plasma treatment (Figure 2c), indicating that cells may uptake or remove extracellular NO generated by plasma.

Similarly, intracellular NO levels in plasma-treated cells were increased by approximately 1.8-fold (1.75±0.20) or 2.3-fold (2.28±0.07) in 1 h and in 18 h following air plasma treatment, respectively, compared with those in non-treated cells. The elevated intracellular NO level was maintained until 24 h (data not shown) following plasma treatment and recovered nearly to the untreated basal level in 48 h (Figure 2d and Figure S1b), similar to ROS levels after plasma treatment (Figure 2b). Air plasma induced a greater and more durable increase in NO than that induced by H_2_O_2_ (Figure 2d and Figure S1b–c). Taken together, these data suggest that plasma treatment induces the generation of extracellular and intracellular NO.

**Figure 3:**
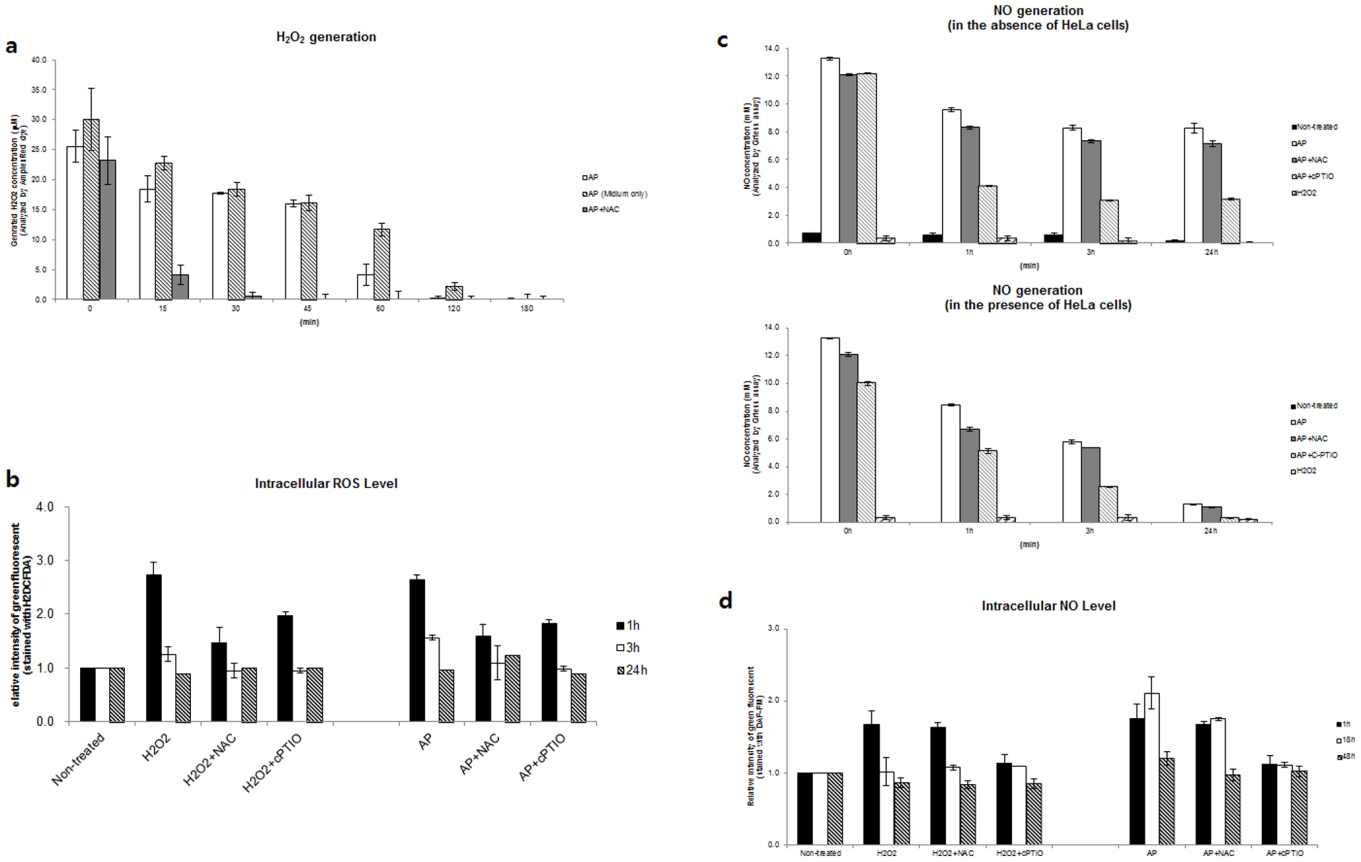
Treatment of antioxidants reduced level of extra- and intracellular ROS/RNS generated by air plasma. (a) Levels of extracellular H_2_O_2_ were determined in culture or non-culture (Medium only) supernatants with air plasma treatment in the presence (AP + NAC) or absence (AP) of the antioxidant NAC (*n* = 5). NAC was added 1h prior to plasma treatment. The culture supernatant was harvested at the indicated times following air plasma treatment. AP 0 (min) indicates supernatant harvested immediately after plasma treatment. (b) Generation of ROS including H_2_O_2_, OH^−^, and •O_2_ in the intracellular matrix following air plasma jet (AP) were determined using the ROS-sensitive probe H_2_DCFDA (*n* = 5). Cells were pretreated with the antioxidants NAC or cPTIO for 1h and then exposed to air plasma (AP+NAC or AP+cPTIO) or H_2_O_2_ (H_2_O_2_+NAC or H_2_O_2_+cPTIO). At indicated times following air plasma treatment, cells were harvested. The fluorescence of untreated cells (Non-treated) was arbitrarily set to 1. (c) Levels of extracellular NO were determined in non-culture (in the absence of HeLa cells, upper) and culture (in the presence of HeLa cells, bottom) supernatants at the indicated times after air plasma jet exposure by the Griess assay (*n* = 10). Medium in the presence or absence of HeLa cells was pretreated with NAC or cPTIO for 1h prior to exposure to plasma or H_2_O_2._ (d) Levels of intracellular NO were evaluated using DAF-FM. Data are shown as the mean ± S.E.M. (*n* = 10).

To support that air plasma generated ROS and RNS levels in mammalian cells, we examined whether antioxidants attenuate the plasma-induced ROS/RNS generation by measuring ROS and RNS levels in HeLa cells following treatment with plasma in the presence of antioxidants. First, we tested whether the well-known thiol antioxidant NAC [24] can affect the concentration of H_2_O_2_ in culture supernatants following air plasma treatment (Figure 3a). In combination with the plasma jet, HeLa cell culture or medium only was pretreated with NAC. NAC induced the rapid decrease of H_2_O_2_ to approximately less than 4.13±1.67 μM in 15 min following the plasma treatment, similar to levels observed in 2 hr after plasma treatment without NAC (Figure 3a), suggesting that NAC can alleviate the air plasma-induced H_2_O_2_ generation. We next examined whether antioxidants may mitigate the air plasma-induced generation of intracellular ROS (Figure 3b). To do this, we evaluated ROS levels following pretreatment with antioxidant NAC or cPTIO and exposure to air plasma. Both NAC and cPTIO could attenuate the generation of intracellular ROS induced by air plasma (Figure 3b). Interestingly, the clear attenuation of intracellular ROS generation by cPTIO, an effective antioxidant of RNS such as nitric oxide (NO^•^), led to speculation that air plasma can generate RNS as well as ROS and the intracellular generation of ROS may be linked to the generation of RNS.

We further examined whether antioxidants may mitigate the air plasma-induced generation of RNS. We found that the NO antioxidant cPTIO promoted decreases in the extracellular NO level induced by plasma in both culture supernatant and non-culture medium (Figure 3c). In contrast, NAC had little effect on the plasma-induced extracellular NO levels in both culture and non-culture medium (Figure 3c). Consistent with the extracellular NO results, c-PTIO but not NAC could significantly abate the generation of intracellular RNS by air plasma (Figure 3d and Figure S1b). The specific attenuation of the air plasma-induced RNS by cPTIO (the effective RNS antioxidant) and attenuation of the H_2_O_2_-induced RNS by both NAC (the effective ROS antioxidant) and cPTIO (Figure 3d) suggest that air plasma can generate RNS directly but the H_2_O_2_-induced RNS generation may be mediated via pathway(s) involving ROS intermediates. Taken together with the ROS and RNS generation data (Figures 2–3), our findings indicate that air plasma can generate extracellular and intracellular ROS and RNS in culture supernatants and the cellular environment.

### Air plasma activates JNK and p38 MAPK-mediated signaling pathway

**Figure 4:**
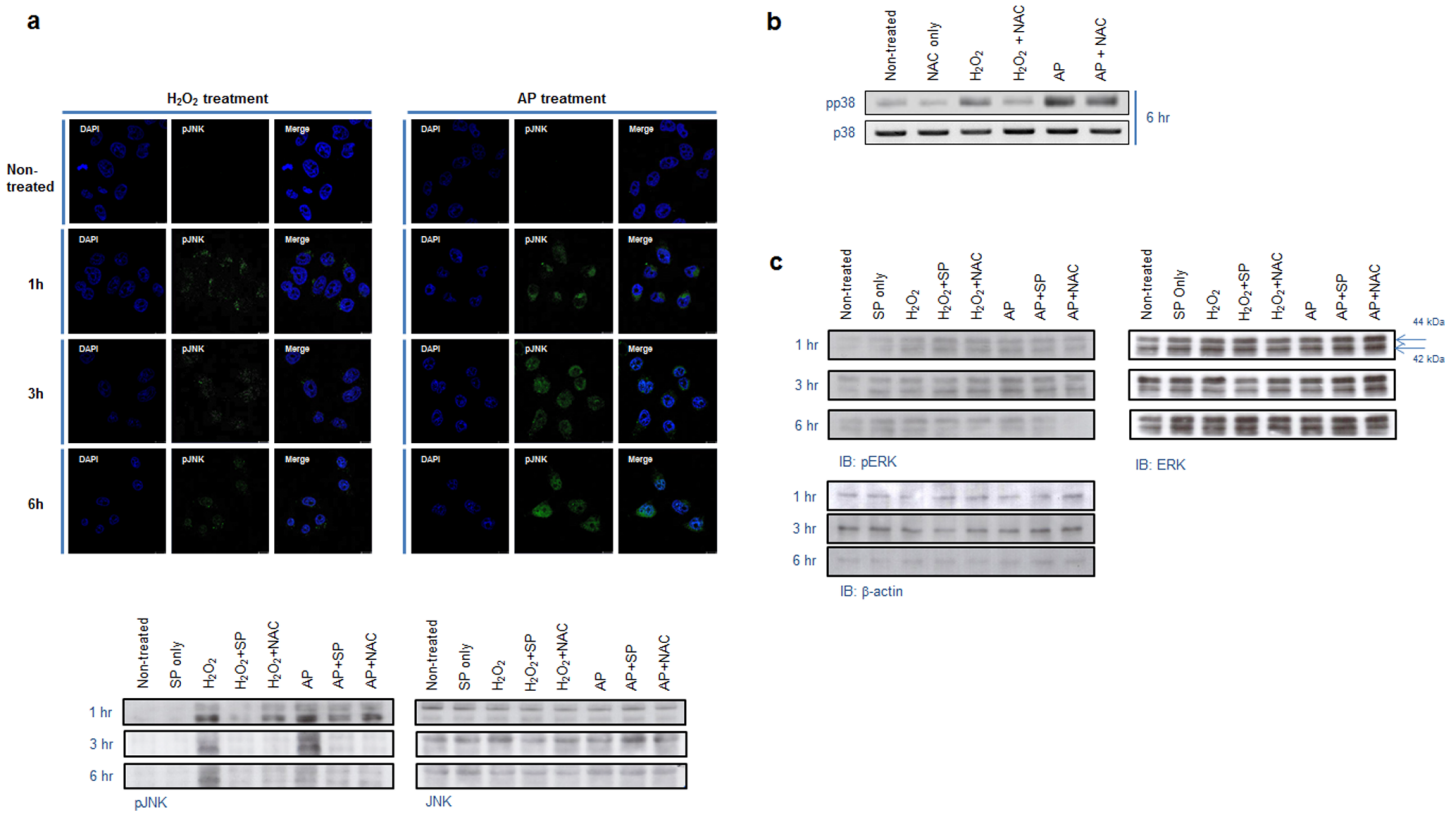
Air plasma-induced phosphorylation of JNK and p38. (a) Phosphorylation of JNK following treatment with air plasma jet or H_2_O_2_ was analyzed via immunofluorescence staining and immunoblotting using anti-phospho-JNK antibody. The equivalent amount of total JNK proteins is shown as a quantitative loading control for JNK phosphorylation. (b) Phosphorylation of p38 induced by air plasma was visualized by immunoblotting using anti-phospho-p38 antibody. The comparable total p38 proteins are shown as a loading control for p38 phosphorylation. (c) Variation of phosphorylation status of ERK was not detected following treatment with air plasma or H_2_O_2._

To determine whether ROS and RNS generated by air plasma can act as signaling molecules and what signal pathways are involved in this transduction, we searched for MAPK cascades that are known to be implicated in signaling pathways in response to oxidative stresses such as ROS, RNS, UV, and ionizing irradiation. To do this, we tested whether the ROS/RNS generated by air plasma induces JNK, p38, and/or ERK activation. Immunofluorescence and immunoblotting analyses were performed to evaluate the phosphorylation of these kinases induced by H_2_O_2_ and its activation in response to air plasma treatment (Figure 4 and Figure S2). Exposure to air plasma highly induced activation of JNK, compared with its modest activation by H_2_O_2_ (Figure 4a and Figure S2a), suggesting that signaling may be evoked by air plasma and may consequently activate a JNK-mediated signal transduction pathway. When cells were treated with plasma or H_2_O_2_ in the presence of an inhibitor of JNK (SP600125), JNK activation was abrogated as expected, in 1h following treatment with plasma or H_2_O_2_ (Figure 4a and Figure S2a). Also, NAC was able to mitigate JNK activation by plasma or H_2_O_2_ (Figure 4a, low panel), indicating that some type of oxidative stress signal may be evoked by the plasma.

Next, we examined the activation of p38 by plasma and whether NAC could affect the p38 activation induced by plasma. In a manner analogous to JNK, air plasma evoked the phosphorylation of p38 and this plasma-induced phosphorylation was partially ameliorated by NAC, indicating that the air plasma-induced signal can activate the p38-mediated signal pathway and may be a kind of oxidizing signal (Figure 4b and Figure S2b). In contrast, plasma had little effect on the phosphorylation of ERK1/2 (Figure 4c). These data, together, suggest that air plasma can evoke intracellular signals and sequentially activate intracellular signal transduction pathways mediated via JNK or p38.

### Air plasma induces mitochondrial ROS accumulation and mitochondrial dysfunction

**Figure 5:**
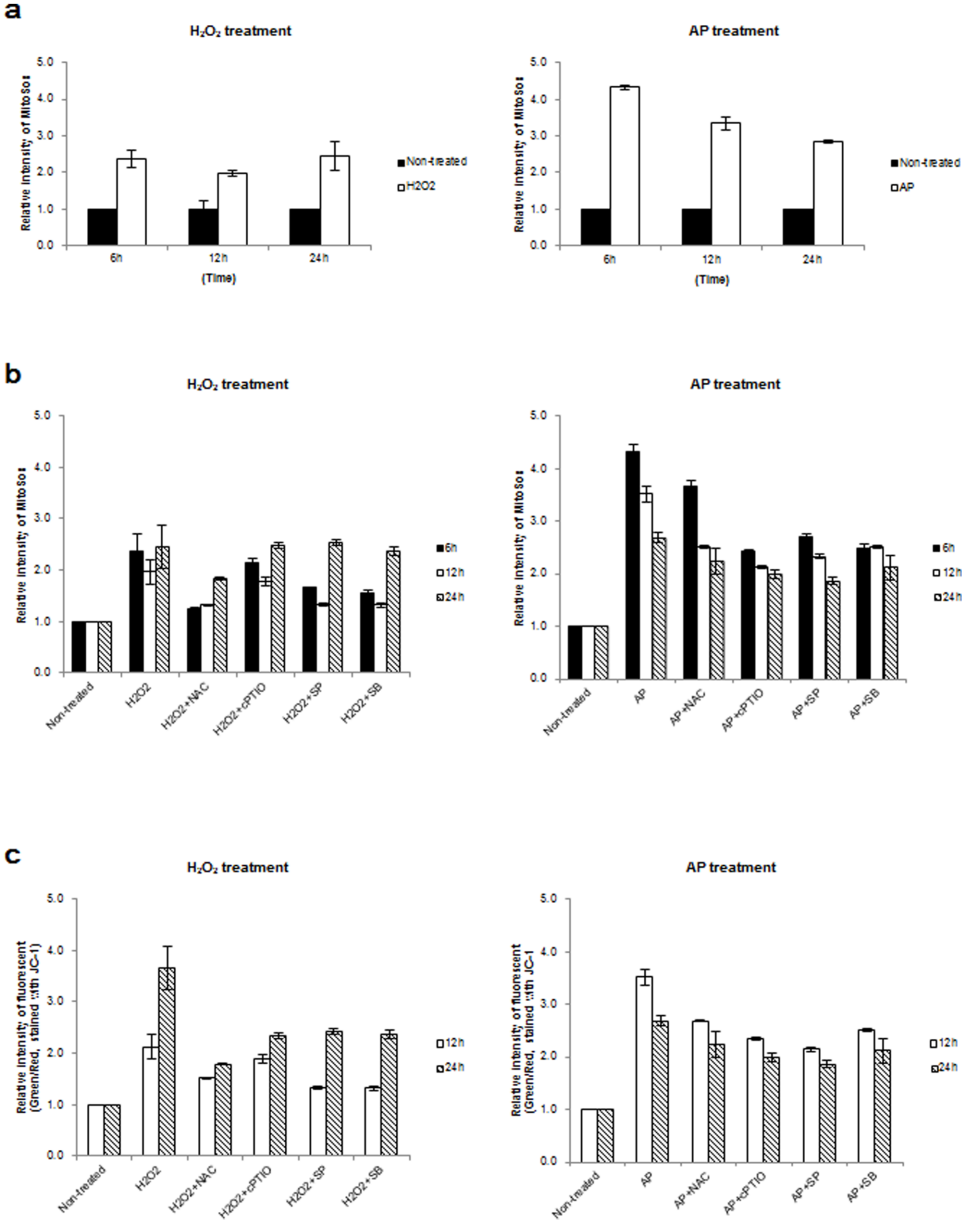
Collapse of the mitochondrial transmembrane potential (Δψm) following plasma treatment. (a) Mitochondrial ROS production was evaluated using the mitochondria-specific probe Mitosox following H_2_O_2_ or air plasma treatment. The mitochondrial ROS in untreated cells (Non-treated) was arbitrarily set to 1. The cells was harvested at the indicated times following air plasma treatment and analyzed by flow cytometry. (b) Cells were pretreated with the antioxidants (NAC or cPTIO) or kinase inhibitors (SP600125 or SB203580) for 1 h and then exposed to air plasma (AP+NAC, AP+cPTIO, AP+SP or AP+SB) or H_2_O_2_ (H_2_O_2_+NAC, H_2_O_2_+cPTIO, H_2_O_2_+SP or H_2_O_2_+SB). Cells were harvested at indicated times following plasma treatment, and levels of mitochondrial ROS were measured (*n* = 5). The fluorescence of untreated cells (Non-treated) was arbitrarily set to 1. (c) The mitochondrial membrane potential was evaluated using the mitochondria-specific probe JC-1 with or without air plasma or H_2_O_2_, (*n* = 5). The fluorescence in untreated HeLa cells was arbitrarily set to 1.

Mitochondria are a major source of cellular ROS generation [25] and oxidative stress including H_2_O_2_ can initiate the collapse of the mitochondrial transmembrane potential (Δψm) [26]. In a previous report, we found that the collapse of the mitochondrial transmembrane potential (Δψm) is induced by N_2_ or air plasma [2]. To see whether plasma can induce mitochondrial ROS accumulation, we measured mitochondrial superoxide following plasma treatment using MitoSox, a fluorogenic dye for the selective detection of superoxide in the mitochondria of living cells (Figure 5a). Mitochondrial superoxide in cells increased upon treatment with air plasma or H_2_O_2_. Furthermore, the level of mitochondrial superoxide in cells treated with air plasma (4.32±0.05) was approximately 2-fold higher than in H_2_O_2_-treated cells (2.36±0.23) at 6 h post-treatment with plasma (Figure 5a). After this time (i.e., 6 h post-treatment), the accumulated mitochondrial superoxide in plasma-treated cells decreased, while the accumulated superoxide in the mitochondria of H_2_O_2_-treated cells persisted until 24 h (2.45±0.39) (Figure 5a). These results suggest that air plasma can induce the accumulation of ROS including superoxide in mitochondria.

In addition, we examined whether antioxidants can attenuate the mitochondrial superoxide accumulation induced by air plasma. NAC or cPTIO was able to abate the production of mitochondrial superoxide (Figure 5b). As air plasma activates JNK and p38-mediated signaling pathways (Figure 4a–b and Figure S2), the possibility that the plasma-activated signal transduction processes are involved in the mitochondrial ROS accumulation was investigated. We found that the kinase inhibitors SB203580 and SP600125 were able to undermine plasma-induced mitochondrial superoxide generation (Figure 5b), implying that plasma can induce ROS production in mitochondria in a JNK- and p38-dependent manner.

Because air plasma induced the generation of cellular ROS/RNS (Figure 2) and mitochondrial ROS (Figure 5a) and the collapse of the mitochondrial transmembrane potential (Δψm) [2], we examined whether ROS/RNS could be linked to the air plasma-induced mitochondrial dysfunction by treating HeLa cells with air plasma in the presence of the antioxidants NAC or cPTIO. We monitored the mitochondrial transmembrane potential (Δψm) following plasma treatment in the absence or presence of NAC or cPTIO, using the mitochondria-specific probe JC-1. Air plasma or H_2_O_2_ treatment induced increasing intensity of green/red fluorescence (approximately 3.3-fold or 2.1-fold at 12 h and 2.9-fold or 3.6-fold at 24 h, respectively), confirming that both plasma and H_2_O_2_ can induce a decrease in Δψm (Figure 5c). NAC or cPTIO showed partially ameliorating effects on the decrease in Δψm induced by plasma or H_2_O_2_ treatment (Figure 5c), suggesting that the air plasma-induced ROS/RNS may play a role in mitochondrial dysfunction. Because the signal transduction processes underlying the plasma-induced decrease in Δψm are unknown, we determined whether inhibitors of JNK and p38 could mitigate the plasma-induced mitochondrial damage. In a similar manner, inhibitors of JNK and p38 (SP600125 or SB203580) were able to attenuate the effects of plasma and H_2_O_2_ on the Δψm decrease, even if not resulting in normal Δψm (Figure 5c). These results suggest that air plasma produces ROS/RNS, leading to activation of signal transduction via JNK or p38. Simultaneously, ROS/RNS appear to induce the collapse of the Δψm, which is partially mediated through JNK and p38 signal transduction

### ROS/RNS generation induces plasma-induced apoptotic cell death via activation of cellular JNK and p38-mediated signal transductions and mitochondrial dysfunction

**Figure 6:**
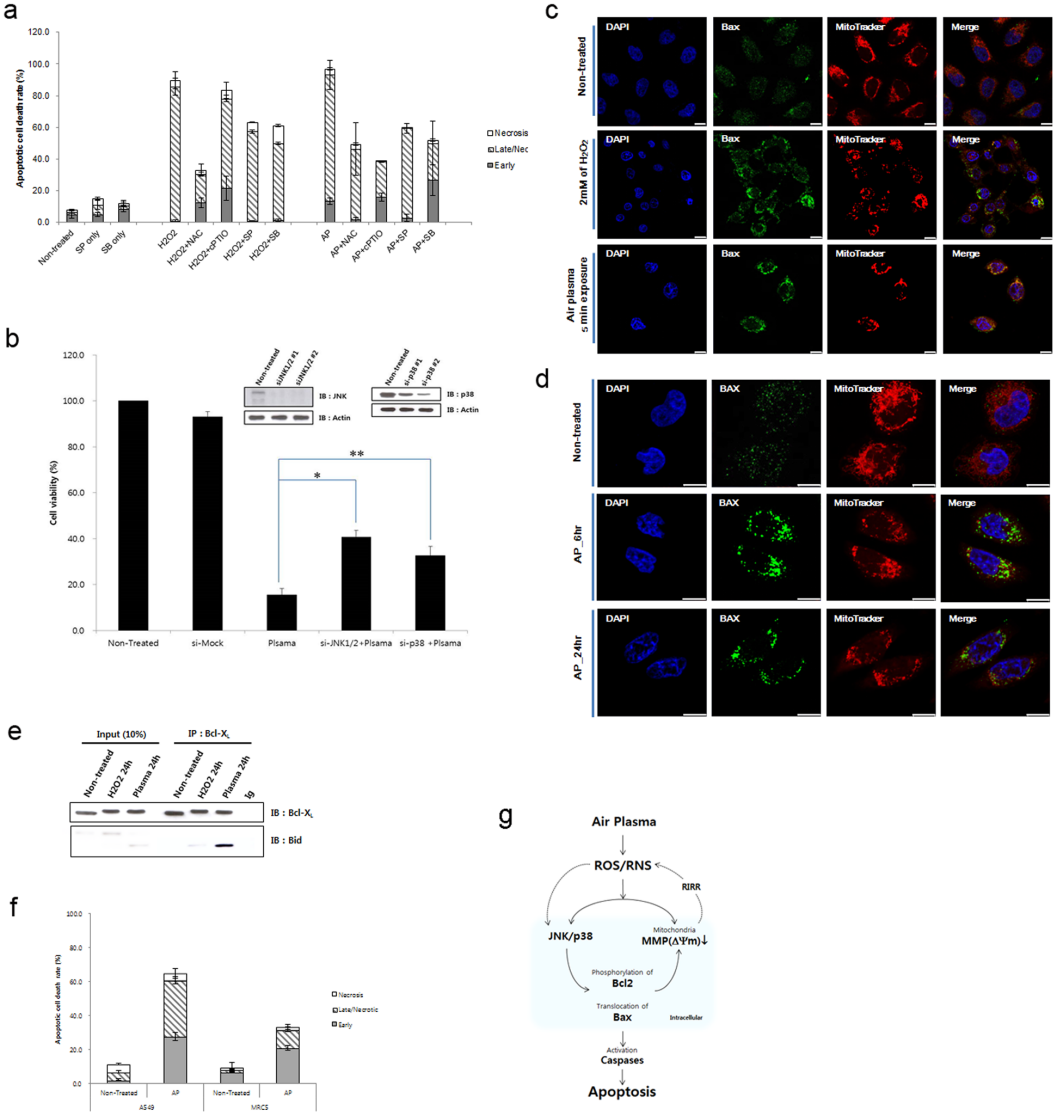
Air plasma induces cell death by activation of JNK and p38 via generating ROS and RNS. (a) Antioxdizing agents (NAC and cPTIO) or kinase inhibitors (SP600125 and SB203580) partially rescued plasma-induced cell death. Data are shown as the mean ± S.E.M. (*n* = 10). (b) Plasma-induced cell death was partially abrogated in JNK1/2 (siJNK1/2) or p38 (sip38) knockdown cells. To monitor plasma-induced cell death, ATP-based cell viability assay was performed in the presence of control, JNK1/2, or p38 siRNA. Data are shown as the mean ± S.E.M. in triplicate from three independent experiment (*n* = 3). Cell viability of untreated HeLa cell population was arbitrarily set to 100%. Immunoblotting with anti-JNK and p38 antibodies was done to confirm knockdown. *p<0.01, **p<0.05. (c)–(d) Air plasma induced Bax translocation to the mitochondria. The plasma- or H_2_O_2_-treated cells were further incubated for 6–24 h. After 6–24 h incubation, cells were fixed by 3.7% formaldehyde. Bax (green) was stained anti-bax antibody and MitoTracker was used for staining of mitochondria (Red). Bax (green) and mitochondrial (red, MitoTracker) fluorescence were assessed, 6 h (d) and 24 h ((c) and (d)) after exposure to air plasma for 5 min by fluorescence confocal microscopy. Bax was diffusely distributed in untreated cells (non-treated). However, after treatment with plasma, Bax was localized to mitochondria, based on the overlap of the Bax and MitoTracker fluorescence images (Merge, yellow). DAPI was used for nuclear staining (blue). White bar was mean magnification of image (10 μM). (e) Bid formed a proapoptotic complex with Bcl-xL following plasma treatment. (f) Air plasma induced differential cell death in human lung adenocarcinoma A549 and normal lung fibroblast MRC5 cell lines. A549 and MRC5 cells were treated with air plasma jets and then incubated further for 24 h. After harvesting and staining cells with anti-annexin V-FITC and PI, cell death was evaluated by flow cytometry. The values represent the mean (s.e.m) from three independent experiments. (g) A proposed model for air plasma jet-induced apoptosis in HeLa cells through ROS/RNS production, trespassing on cells, activation of signal transduction pathways, and mitochondrial damage.

To determine air plasma-induced cell death, we first tested the possibility that the ROS/RNS were responsible for air plasma-induced cancer cell death by treating HeLa cells with air plasma in the presence of the antioxidants NAC or cPTIO. Pretreatment with NAC and cPTIO attenuated plasma-induced cell death (49.5%±2.2% and 38.86%±4.2%, respectively, Figure 6a and Figure S3), corroborating that ROS/RNS is implicated in plasma-induced cell death. Because there is no good control for RNS similar to the use of H_2_O_2_ as a canonical control for ROS, we also tested whether NaNO_3_ could induce apoptosis, plausibly involving NO and RNS generated from NaNO_3_. NaNO_3_ promoted cell death at higher concentrations than did H_2_O_2_ (Figure S3–S4). According to this result, the reason NaNO_3_ does not induce effective cell death is that it fails to generate effective RNS/ROS unlike air plasma or H_2_O_2_ (Figure S4).

To investigate role of the JNK and p38 MAPK pathways in air plasma-induced apoptotic cell death, we employed their specific inhibitors, SP600125 (inhibitor of JNK) and SB203580 (inhibitor of p38 MAPK). When cells were pretreated with SP600125 or SB203580 prior to air plasma jet exposure, the apoptotic cell death population was reduced to 59.3%±2.3% and 52.0%±9.7%, respectively, compared with cell death induced by the plasma jet alone (85.4±3.4%) (Figure 6a and Figure S3), suggesting that signal transduction via JNK and p38 is partially involved in plasma-induced cell death. To get more insight into the roles of JNK and p38 during the plasma-induced cell death, cell viability of JNK- or p38-depleted cells was analyzed following plasma treatment by measuring ATP from viable cells. We found that the plasma-induced decrease in cell viability was attenuated by JNK1/2 siRNA or p38 siRNA (Figure 6b). Consistent with the abrogative effects of inhibitors of JNK or p38 on the plasma-induced cells death, cell viability in the presence of JNK1/2 or p38 siRNA was higher (40.85±2.38% or 32.68±3.91%, respectively), when compared with viability of non-depleted cells (15.47±2.81%) after plasma treatment. These results suggest that the plasma induces cell death in a JNK- and p38-depedent manner.

A transient increase in mitochondrial membrane hyperpolarization following exposure to oxidative stress including H_2_O_2_ initiates the decreased mitochondrial membrane potential [27]–[31], leading to the mitochondrial translocation of Bax and Bid and cytochrome c release [32]. We asked whether air plasma could stimulate Bax translocation to the mitochondria, thereby activating the cell death pathway involving mitochondrial dysfunction. While Bax was diffusely distributed in non-treated control cells, it was localized to mitochondria after treatment with plasma exposure. Non-treated control cells typically displayed a diffuse, cytosolic pattern of Bax localization (Figure 6c–d). However, air plasma exposure produced significant changes in the distribution of fluorescence that was detected 6 h after treatment and involved a change from diffuse, cytosolic distribution to a punctate pattern of Bax, which was maintained up to 24 h after plasma treatment (Figure 6d), suggesting that air plasma induced Bax translocation to the mitochondria. To investigate Bcl-xL-Bax pathway, we determined whether prosurvival Bcl-xL could form a proapoptotic complex with proapoptotic BH3 subfamily protein such as Bid and Bim after plasma treatment. Thus, we performed a coimmunoprecipitation assay. As shown in Figure 6e, Bcl-xL interacted with Bid after plasma treatment but they did not interact in the non-treated cells, suggesting that plasma induces inactivation of Bcl-xL via interaction with Bid, which would lead to pore formation at the mitochondria and release of cytochrome c. In addition, phosphorylation of Bcl-2 and Bcl-X_L_ was observed following plasma treatment (Figure S5), further indicating the plasma-induced proapoptotic events. Since Bcl-xl-Bid interaction can promote the release of cytochrome, we examined whether plasma treatment could induce cytochrome c release from mitochondria. We found that cytochrome c was released from the mitochondria, following plasma treatment (Figure S6). Together, these findings indicate that Bax translocates to the mitochondrial outer membrane and then promotes interaction of Bcl-xL with Bid (Figure 6), thereby triggering mitochondrial pore formation and cytochrome c release (Figure S6), in addition to mitochondrial ROS accumulation (Figure 5a) and collapse of the mitochondrial transmembrane potential (Δψm) (Figure 5b). Taken together, our observations have implications in identifying the causal relationships among ROS/RNS production, JNK/p38 activation, mitochondrial dysfunction, and cell death induced by air plasma.

We further tested whether air plasma may exhibit cancer-selective cytotoxicity by employing human alveolar epithelial adenocarcinoma A 549 and human fetal lung fibroblast MRC5 cells. Plasma induced cell death highly by 64.70±5.46% in A549 lung cancer cells but only by 33.20±3.26% in MRC5 (Figure 6f and Figure S7). This result suggests that air plasma induces cell death more effectively in cancerous lung cells than in normal lung cells, and further disclose its cancer-selective proapoptotic potential.

## Discussion

### As a key effector for cancer cell death, ROS/RNS generated by air plasma

By demonstrating that air plasma produces extracellular ROS and RNS and the generated extracellular ROS/RNS appear to translocate into the cells and induce intracellular and mitochondrial ROS/RNS production, this study, in conjunction with previous studies [2], [33], explains what components and how air plasma induces cancer cell death. Our findings indicate air plasma generates a variety of ROS (H_2_O_2_, O_2_^−^ and ^•^O_2_) and RNS (NO, ONOO^−^) (Figure 1, 2 and 3) and these radicals induce an apoptotic signal cascade (Figure 6d) by inducing MAPK signaling including JNK and p38 MAPK (Figure 4), with simultaneous mitochondrial dysfunction (Figure 5, 6). Because the reaction rate of ROS and RNS is rapid (theoretically nanosecond level), it is puzzling that ROS and RNS generated by air plasma first induce mitochondrial dysfunction before activation of cellular signal transduction, rather than in the reverse order or simultaneously. Antioxidants and kinase inhibitors can attenuate induction of ROS/RNS and mitochondrial ROS, mitochondrial dysfunction, and apoptotic activity induced by plasma, supporting the crucial role of ROS/RNS in the plasma-induced anticancer effects.

### Targeting cancer cells with a radical approach by atmospheric air plasma

Plasma has just recently been emerging as anti-cancer therapeutic agents given its cancer-cell-apoptotic activities [2], [14]–[19]. While cancer-cell-selective killing by air plasma has been observed [2, unpublished data], exactly how and why cancer cells are sensitive to plasma remain unknown. We have recently suggested a link between generation of ROS and cancer-cell death following plasma treatment has been suggested [2]. Targeting cancer cells through ROS-mediated mechanisms has become an attractive strategy for the effective and selective cancer treatment by exploiting the aberrant redox characteristics of cancer cells [34]. Our study corroborates that ROS/RNS induced by air plasma effectively target cancer cells via mitochondrial dysfunction and activation of oxidative stress signaling pathways. In conjunction with the radical therapeutic approach, air plasma may have potentials for anti-cancer therapeutics.

Our findings on the mechanism of air plasma action have implications for ROS/RNS-mediated pharmacological drug design and therapeutic strategy. In eukaryotic organisms, generation of ROS and RNS is induced by both endogenous and exogenous stimuli. ROS and RNS are generated from diverse sources including mitochondrial electron transport system, endoplasmic reticulum system, and NADPH oxidase complex. The main sources, mitochondria, sense various external signals and stresses such as TNFα, hypoxia, radiation, chemical agents, and oxidative stresses to induce mitochondrial ROS production. It has been suggested that these stress-induced ROS produced by mitochondria play roles in neighboring mitochondrial damage and cell death, which is termed ROS-induced ROS release (RIRR) [35], via a positive feedback loop [36]. Thus, mitochondria can become an amplifier and also a key target of ROS/RNS generated by air plasma. As shown in this study, this is indeed the case. Mitochondrial ROS generation initiated by air plasma-induced ROS/RNS was abruptly elevated by 6 h following air plasma treatment and then the level gradually decreased (Figure 5a), suggesting an acute response rather than a typical positive feedback loop (RIRR hypothesis) triggered by external stimuli including TNFα or a single ROS inducer such as H_2_O_2_. Mitochondrial ROS heavily accumulated in response to air plasma and the initial high mitochondrial ROS level decreased gradually (Figure 5a). However, H_2_O_2_ treatment induced consistent ROS level (Figure 5a). Based on our observations, mitochondria seem to respond differentially to the extracellular ROS/RNS generated by air plasma. We do not have a good explanation for this. However, the identity of the ROS/RNS and probably their composition are very different between air plasma and endogenous (for example, mitochondrial) origins or external single-component stimuli such as H_2_O_2_.

Another possible explanation for the differential mitochondrial response to air plasma is that most mitochondria are assaulted by air plasma and little remain available for the RIRR loop, while part of mitochondria are damaged by endogenous origins or external single-component stresses. Our data suggest that air plasma composed of various reactive species can severely damage most mitochondria simultaneously (Figure 5), while TNFα or H_2_O_2_ can cause local or specific damage in some mitochondria. Since no normal mitochondria were left over when cells encountered ROS/RNS induced by air plasma, the released ROS cannot attack mitochondria even if mitochondrial ROS production and release (RIRR) were to occur. Therefore, air plasma, which generates a combination of various ROS and RNS, can induce acute mitochondrial ROS accumulation and cell death in a snapshot mode, while other external signals or single-component stress can induce the typical positive feedback loop.

Since the levels of ROS in cancer cells are close to the limit at which cell death occurs, and the sources of ROS production in cancer cells are different from those in robust cells [35], ROS have been explored as anti-cancer therapeutic drugs. The possibility has been raised of ROS upregulation by inhibitors of antioxidant enzymes or by ROS inducers, which lead to further oxidative stress and preferentially give rise to cancer cell death, as anti-cancer therapeutic agents [37]. In this regard, our results may have clinical implications for cancer treatment using plasma. The increase in ROS/RNS and the resulting mitochondrial dysfunction induced by plasma (Figure 6g) may partially ameliorate the clinical course of cancers. As such, plasma may be a potential drug for the treatment of cancer or ROS homeostasis to prevent ROS-related disease, in addition to antiseptics.

## Materials and Methods

### Reagents

Hydrogen peroxide (H_2_O_2_), N-acetyl-cysteine (NAC), N-(1-naphthyl)ethylenediamine dihydrochloride, sulfanilamide, 2-methyl-2-thiopseudourea hemisulfate salt, sodium nitrite, orthophosphoric acid, and 4′,6-diamidino-2-phenylindole (DAPI) were purchased from Sigma-Aldrich Chemical Co. (Saint Louis, MO). 5,5′,6,6′-tetrachloro-1,1′,3,3′-tetraethyl-benzimidazolylcarbocyanine chloride (JC-1), 2-(4-carboxyphenyl)-4,4,5,5-tetramethylimidazoline-1-oxyl-3-oxide (cPTIO), ROS-sensitive dichlorodihydrofluorescin diacetate (H_2_DCFDA), 4-amino-5-methylamino-2′,7′-difluorofluorescein diacetate (DAF-FM diacetate), and MitoTrackerTM were purchased from Invitrogen (Carlsbad, CA). SP600125 and SB203580 were obtained from Calbiochem (San Diego, CA). Fluorescent mounting medium was purchased from Dako (Carpinteria, CA). Primary antibodies used in this study include anti-Bax, anti-JNK, anti-extracellular signal-related kinase (ERK), anti-phospho-ERK, anti-p38, anti-phospho-p38, anti-caspase 3, anti-bid, anti-Bcl-xL, anti-phospho-Bcl2, anti-cytochrome C, (Cell Signaling Technology, Beverly, MA), and anti-phospho-JNK (Invitrogen, CA), anti-actin and anti-COX IV (Abcam, Cambridge, UK), and anti-PARP (BD Bioscience, San Jose, CA) antibodies. Horseradish peroxidase (HRP)-conjugated and fluorescently labeled Alexa488- and Alexa546-conjugated antibodies (Invitrogen) were used as secondary antibodies.

### Design and fabrication of air microplasma jet system

In this study, we used a homemade plasma jet system with a micro-nozzle array to treat cervical cancer HeLa cells (Figure 1a). The plasma jet nozzle consists of a plasma generation module and cases. The plasma generation module consists of an anode, a porous ceramic insulator, and a cathode. The anode is fabricated by means of conventional photolithography and Ni electroplating. Two hundred holes are arrayed along concentric circles on the anode, and the diameter and the depth of each hole are 100 μm and 60 μm, respectively. Gas is supplied through a gas inlet and the gas is ionized in the pores of the ceramic insulator by an electric field between 2 electrodes. The electric field is formed by 20 kVp-p at 20-kHz bias. The plasma generated in the ceramic pores is ejected to the atmosphere through the anode holes (Figure 1b).

### Cell culture and treatment of cells with air plasma jet

Human cervical cancer HeLa cells (ATCC CCL-2), lung cancer A549 cells, and normal lung MRC5 cells were cultured in DMEM, supplemented with 10% FBS for 24 hr. Cells grown to 90% confluence were exposed to an air plasma jet from the device located 3.8 cm away, for 5 min. The ROS and RNS scavenging agents, 4 mM NAC or 100 μM carboxyl-PTIO (cPTIO), were added 1 hr prior to plasma treatment. Cells were exposed to the plasma jet, immediately after adding 25 μM SP600125 (JNK inhibitor) or 10 μM SB203580 (p38 kinase inhibitor).

### ROS and RNS detection assay

Levels of H_2_O_2_ and ROS were measured using the Amplex UltraRed hydrogen peroxide assay kit (Invitrogen) and H_2_DCFDA, respectively [2]. To quantitate ROS, cells were incubated with 5 μM H_2_DCFDA for 30 min. Florescence intensity was measured in a microplate reader (Bio-Rad, Hercules, CA) at 530/590 nm (Amplex UltraRed) or 485/535 nm (H_2_DCFDA), according to the manufacturer's protocol. The relative ROS level was calculated in terms of arbitrary fluorescence units. RNS generation in the culture supernatant by plasma was detected by the Greiss assay [38]. To detect the intracellular generation of NO, cells were incubated with 10 μM DAF-FM at 37°C for 30 min. Cells were analyzed by flow cytometry (BD FACSAria^TM^ III, BD Bioscience, San Jose, CA).

### Immunoblotting, immunofluorescent staining, and coimmunoprecipitation

Following plasma treatment, cells were lysed in RIPA buffer (25 mM Tris-HCl, pH 7.6, 150 mM NaCl, 1% NP40, 1% sodium deoxycholate, 0.1% SDS) supplemented with protease (protease inhibitor cocktail set; Calbiochem, San Diego, CA) and phosphatase inhibitors for western blotting. Cell lysates were immunoblotted using anti-phospho-JNK (1∶2000), anti-p38 (1∶000), anti-ERK (1∶1000), anti-phospho-ERK (1∶000), anti-phospho-p38 (1∶1000), anti-PARP (1∶1000), and Bid (1∶1000) antibodies. For immunofluorescence staining, cells were incubated with MitoTracker, followed by staining with anti-Bax, anti-cytochrome or anti-COX VI primary (1∶250) and Alexa488-conjugated secondary antibodies. The cell nuclei were strained with DAPI. Samples were analyzed under an LSM700 confocal laser-scanning microscope (Carl Zeiss, Thornwood, NY). For coimmunoprecipiation experiment, immunoprecipitation was performed using anti-Bcl-xL (1∶250) and immunoblotting was done with anti-Bid antibody (1∶1000).

### Quantification of mitochondrial membrane permeability (MMP)

Plasma-treated cells (1×10^5^) were further incubated for 12–24 hr to measure mitochondrial membrane permeability (MMP). After 24 h incubation, cells were stained with 2.5 μM JC-1, a lipophilic cationic fluorescence dye with a dual emission wavelength for 30 min, in order to analyze the depolarization of the mitochondrial transmembrane potential (Δψm). Increased mitochondrial membrane potential has been shown to increase JC-1 fluorescence at 530 nm (FITC-A), which corresponds to its monomeric form, and to reduce JC-1 fluorescence at 590 nm (PI-A), which corresponds to its dimeric form. Following staining, cells were analyzed using flow cytometry. Data are shown as the mean ± S.E.M. (*n* = 10).

### Measurement of apoptotic cell death

HeLa cells were treated with air plasma jet for 5 min as indicated and incubated further for 24 hr. To detect plasma-induced apoptosis, cells were stained with Alexa488-conjugated annexin V antibody (Invitrogen) and propidium iodide (PI, BD Bioscience) following the manufacturer's protocol. Following staining, cells were analyzed using flow cytometry. Data are shown as the mean ± S.E.M. (*n* = 7). PARP cleavage and caspase activation were determined as additional apoptotic markers by immunoblotting using anti-PARP or anti-caspase antibody, respectively. Caspase-mediated cleavage of Bid was examined by immunoblotting with anti-Bid anitbody. Cell viability assay was done using the CellTiter-Glo® Luminescent Cell Viability Assay kit (Promega, Madison, WI) that measures the amount of ATP from viable cells according to the manufacturer's protocol (n = 3).

### siRNA construction and transient transfection

Double-stranded JNK1/2 and p38 siRNAs were generated using the pSUPER vector, as described [39]–[40]. Transfections were done using the Effectene Transfection Kit (Qiagen, Carlsbad, CA) according to the manufacturer's instructions.

### Statistical analysis

All of the experimental data were obtained at least in triplicate unless otherwise mentioned and are presented as mean ± standard deviation. Statistical comparison by analysis of variance was performed at a significance level of p<0.05 based on the Student's t-test.

## Supporting Information

Figure S1 **Generation of ROS and RNS by air plasma.** (a) Following the addition of 2 mM H_2_O_2_, levels of H_2_O_2_ in culture or non-culture (Medium) supernatants were evaluated using Amplex UltraRed fluorescent dye. NAC or cPTIO was added 1 h prior to H_2_O_2_ treatment. The culture supernatant was harvested at the indicated times after H_2_O_2_ treatment (H_2_O_2_) or not (Non-treated) (*n* = 5). (b) Time course of generation of intracellular matrix NO induced by air plasma. Data are shown as the mean ± S.E.M. (*n* = 10). The levels of intracellular NO were determined by DAF-FM staining. (c) Time course of generation of intracellular matrix NO induced by H_2_O_2_ for 48 h.
(TIF)

Figure S2 **Air plasma induces phosphorylation of JNK (a) and p38 (b) kinases.** Whole cell lysates of HeLa cells, which were treated by air plasma jet or H_2_O_2_, were subjected to immunoblot analysis to measure phosphorylated JNK (a) and p38 (b) proteins with anti-phospho-JNK or p38 antibodies, respectively. The levels of phosphorylated JNK (a) and p38 (b) proteins in the immunoblots were quantified using Image J software, which is a public domain Java image processing program developed at the National Institutes of Health (http://rsbweb.nih.gov/ij/index.html). The relative intensity of phosphorylated kinase to total kinase proteins from the control sample was arbitrarily set to 1. The representative immunoblots analyzing phosphorylated JNK and p38 proteins are shown in Figure 4 and the levels of phosphorylated JNK and p38 represent the mean (s.e.m) from three separate immunoblots.
(TIF)

Figure S3 **Air plasma-induced apoptosis in HeLa cells, a human cervical carcinoma cell line, as analyzed by flow cytometry.** (a) Cells were treated with H_2_O_2_ and air plasma jets, in the presence or absence of SP600125 and NAC, and then incubated further for 24 h. After harvesting, cells were stained with anti-annexin V-FITC and PI and analyzed by flow cytometry. (b) Air-plasma-induced apoptosis was estimated by PARP cleavage and caspase activation. (c) Caspase-mediated Bid cleavage was observed following air-plasma treatment.
(TIF)

Figure S4 **Dose dependent effect of NaNO_3_, an inducer of NO production, on growth inhibition.** HeLa and Raw264.7 cells were subjected to NaNO_3_ at the indicated concentrations (3–200 mM) and the growth inhibition of cells was quantified by the MTT assay. The growth of untreated cells was arbitrarily set to 100%. Data are shown as the mean ± S.E.M. (*n* = 5).
(TIF)

Figure S5 **Phosphorylation of prosurvival Bcl-2 family proteins after plasma treatment.** Anti-phospho-Bcl2 antibody was used for detecting phosphorylation of Bcl2. When compared with Bcl-xL protein band from non-treated cells, retarded Bcl-xL from plasma-treated cells indicates its phosphorylation following plasma treatment
(TIF)

Figure S6 **Cytochrome c was released from mitochondria after plasma treatment.** (a) Plasma treatment induced cytochrome c release from mitochondria. Cytochrome *c* (green) was stained anti-cytochrome *c* antibody and MitoTracker was used for staining of mitochondria (Red). DAPI was used for nuclear staining (blue). White bar was mean magnification of image (10 μM). (b) Cox IV was stained in mitochondria (red) by anti-Cox IV (green) antibody, without regard to plasma treatment.
(TIF)

Figure S7 **Cancer-selective cytotoxicity by air plasma.** The human lung adenocarcinoma epithelial A549 and normal fibroblast MRC5 cells were treated with air plasma jets and then incubated further for 24 h. After harvesting and staining cells with anti-annexin V-FITC and PI, cell death was evaluated by flow cytometry.
(TIF)
