# Supplementary figures and images for: Targeting Cancer Cells with Reactive Oxygen and Nitrogen Species Generated by Atmospheric-Pressure Air Plasma
Source: PLoS One. 2014 Jan 21;9(1):e86173. doi: 10.1371/journal.pone.0086173 (PMC3897664; doi:10.1371/journal.pone.0086173)

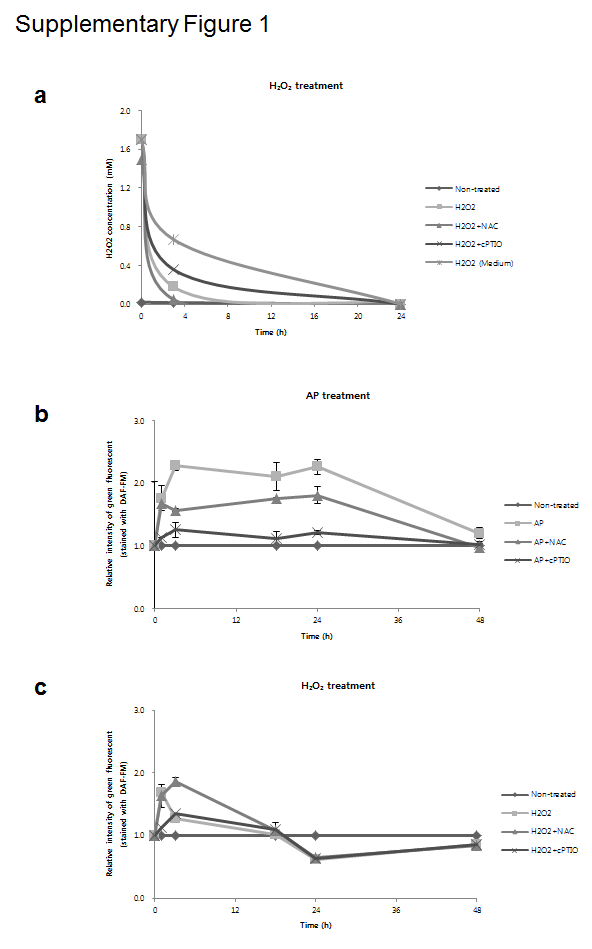

Supplement: Figure S1 — Generation of ROS and RNS by air plasma. (a) Following the addition of 2 mM H2O2, levels of H2O2 in culture or non-culture (Medium) supernatants were evaluated using Amplex UltraRed fluorescent dye. NAC or cPTIO was added 1 h prior to H2O2 treatment. The culture supernatant was harvested at the indicated times after H2O2 treatment (H2O2) or not (Non-treated) (n = 5). (b) Time course of generation of intracellular matrix NO induced by air plasma. Data are shown as the mean ± S.E.M. (n = 10). The levels of intracellular NO were determined by DAF-FM staining. (c) Time course of generation of intracellular matrix NO induced by H2O2 for 48 h. (TIF) [file pone.0086173.s001.tif]

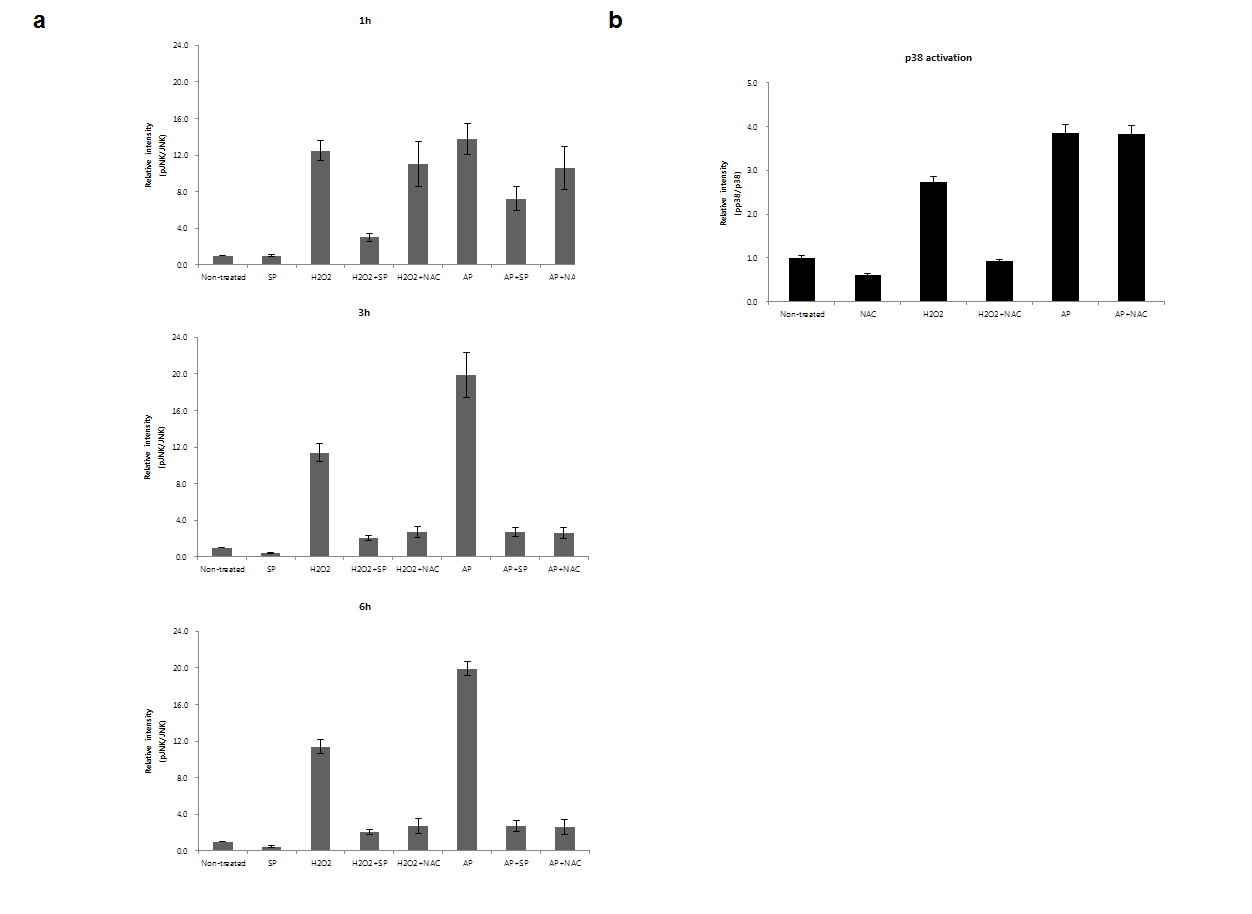

Supplement: Figure S2 — Air plasma induces phosphorylation of JNK (a) and p38 (b) kinases. Whole cell lysates of HeLa cells, which were treated by air plasma jet or H2O2, were subjected to immunoblot analysis to measure phosphorylated JNK (a) and p38 (b) proteins with anti-phospho-JNK or p38 antibodies, respectively. The levels of phosphorylated JNK (a) and p38 (b) proteins in the immunoblots were quantified using Image J software, which is a public domain Java image processing program developed at the National Institutes of Health (http://rsbweb.nih.gov/ij/index.html). The relative intensity of phosphorylated kinase to total kinase proteins from the control sample was arbitrarily set to 1. The representative immunoblots analyzing phosphorylated JNK and p38 proteins are shown in Figure 4 and the levels of phosphorylated JNK and p38 represent the mean (s.e.m) from three separate immunoblots. (TIF) [file pone.0086173.s002.tif]

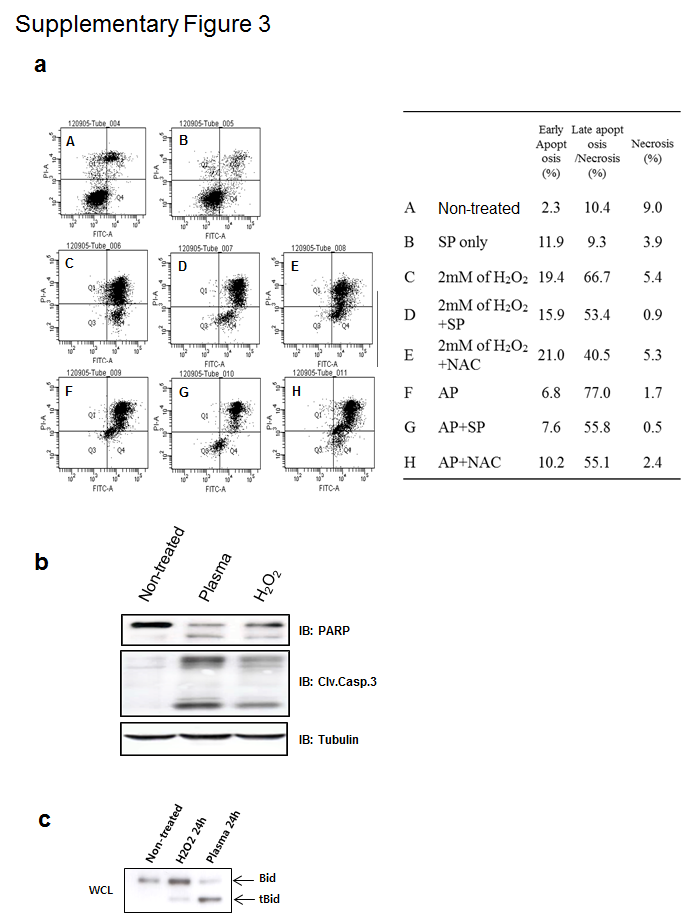

Supplement: Figure S3 — Air plasma-induced apoptosis in HeLa cells, a human cervical carcinoma cell line, as analyzed by flow cytometry. (a) Cells were treated with H2O2 and air plasma jets, in the presence or absence of SP600125 and NAC, and then incubated further for 24 h. After harvesting, cells were stained with anti-annexin V-FITC and PI and analyzed by flow cytometry. (b) Air-plasma-induced apoptosis was estimated by PARP cleavage and caspase activation. (c) Caspase-mediated Bid cleavage was observed following air-plasma treatment. (TIF) [file pone.0086173.s003.tif]

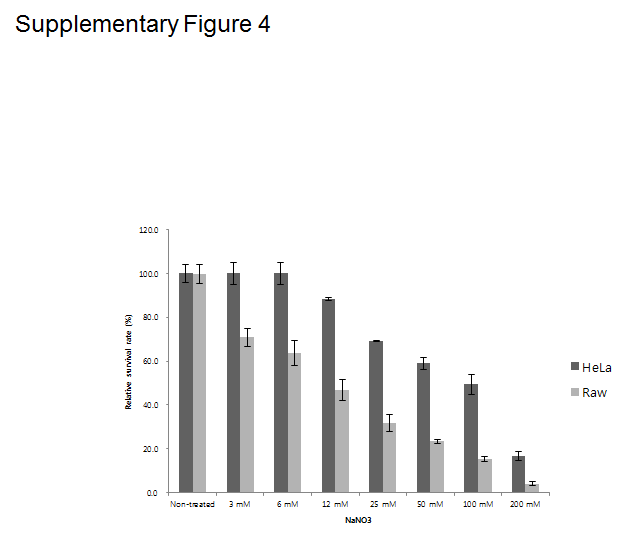

Supplement: Figure S4 — Dose dependent effect of NaNO3, an inducer of NO production, on growth inhibition. HeLa and Raw264.7 cells were subjected to NaNO3 at the indicated concentrations (3–200 mM) and the growth inhibition of cells was quantified by the MTT assay. The growth of untreated cells was arbitrarily set to 100%. Data are shown as the mean ± S.E.M. (n = 5). (TIF) [file pone.0086173.s004.tif]

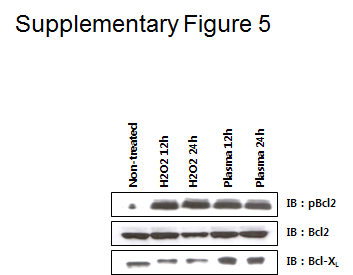

Supplement: Figure S5 — Phosphorylation of prosurvival Bcl-2 family proteins after plasma treatment. Anti-phospho-Bcl2 antibody was used for detecting phosphorylation of Bcl2. When compared with Bcl-xL protein band from non-treated cells, retarded Bcl-xL from plasma-treated cells indicates its phosphorylation following plasma treatment (TIF) [file pone.0086173.s005.tif]

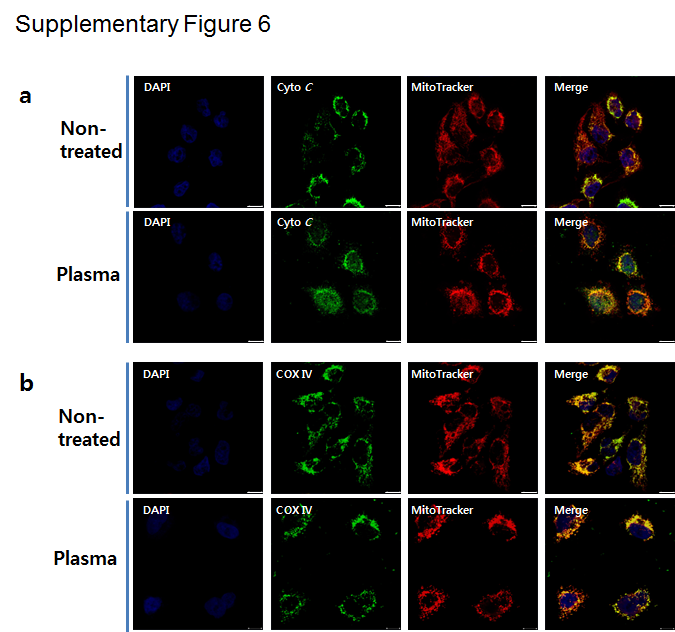

Supplement: Figure S6 — Cytochrome c was released from mitochondria after plasma treatment. (a) Plasma treatment induced cytochrome c release from mitochondria. Cytochrome c (green) was stained anti-cytochrome c antibody and MitoTracker was used for staining of mitochondria (Red). DAPI was used for nuclear staining (blue). White bar was mean magnification of image (10 μM). (b) Cox IV was stained in mitochondria (red) by anti-Cox IV (green) antibody, without regard to plasma treatment. (TIF) [file pone.0086173.s006.tif]

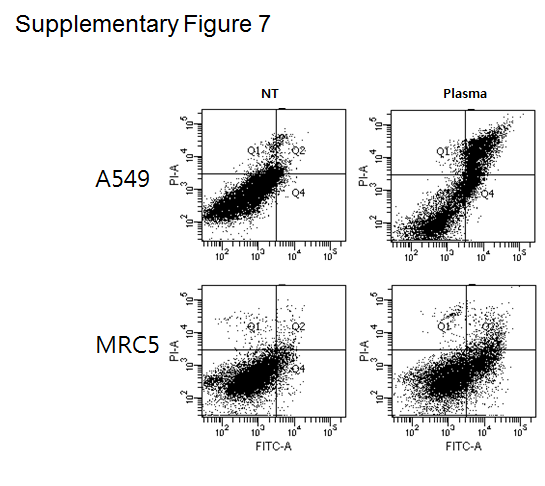

Supplement: Figure S7 — Cancer-selective cytotoxicity by air plasma. The human lung adenocarcinoma epithelial A549 and normal fibroblast MRC5 cells were treated with air plasma jets and then incubated further for 24 h. After harvesting and staining cells with anti-annexin V-FITC and PI, cell death was evaluated by flow cytometry. (TIF) [file pone.0086173.s007.tif]
